# Genetic Diversity Analysis and Identification of Candidate Genes for Growth Traits in Chengkou Mountain Chicken

**DOI:** 10.3390/ijms252312939

**Published:** 2024-12-02

**Authors:** Lingbin Liu, Yi Wang, Yu Huang, Zhen Wang, Qigui Wang, Haiwei Wang

**Affiliations:** 1 Chongqing Academy of Animal Sciences, Rongchang, Chongqing 402460, China; liulb515@163.com (L.L.); zhwang2022@163.com (Z.W.); 2 College of Animal Science and Technology, Southwest University, Beibei, Chongqing 400715, China; hddszxf@163.com (Y.W.); h20001026@email.swu.edu.cn (Y.H.)

**Keywords:** genome-wide association studies (GWASs), candidate genes, growth traits, Chengkou mountain chicken

## Abstract

Growth traits constitute critical factors in the breeding program of broiler chickens. The Chengkou mountain chicken A-lineage (CMC-A) represents a breed specifically bred for meat production. To further explore the growth performance of the CMC-A population, this study conducted whole-genome sequencing on 464 CMC-A roosters to systematically evaluate their genetic diversity. Additionally, runs of homozygosity (ROH) islands and genome-wide association studies (GWASs) were employed to identify the loci and functional genes influencing the growth traits in Chengkou mountain chickens. The results revealed a high level of genetic diversity and low levels of inbreeding in Chengkou mountain chickens. Several genes associated with stress resistance, muscle growth, and fat deposition were pinpointed through ROH island identification. Moreover, 52 SNP loci were detected, along with 71 candidate genes. These findings enhance our understanding of the genetic architecture underlying the growth traits in Chengkou mountain chickens and provide a theoretical foundation for subsequent breeding endeavors.

## 1. Introduction

The Chengkou mountain chicken (CMC) is a local chicken breed in Chengkou County, China, bred by natives for generations under specific environmental conditions. The CMC breed exhibits tolerance to coarse feed, delicious meat quality, highly nutritious meat and eggs, and hardiness, making it a key economic pillar in the area [1]. Similar to chicken breeds in other regions of China, CMC production is constrained by the low growth performance and low meat production. To address this issue, researchers have developed a commercial meat line through breeding, the Chengkou mountain chicken A-lineage (CMC-A), with a significantly improved growth performance. This has further enhanced the economic value of the CMC. However, chicken growth traits are usually highly heritable [2], but will be impacted by many minor-effect alleles, and are difficult and expensive to measure, reducing the potential rate of genetic improvements through traditional breeding methods. This hinders the rapid improvement of production traits, thereby seriously impeding the breeding and industrialization process of efficiently producing CMCs.

As contiguous homozygous segments within the genome of diploid organisms, runs of homozygosity (ROH) [3] can be utilized to assess the inbreeding levels [4] and genetic diversity [5], and to screen candidate genes for selective traits [6]. Genome-wide association analysis (GWAS) is a genome-wide method of searching for associations between genetic variants (SNPs) and complex traits. GWASs are based on the principle of linkage disequilibrium (LD), the idea that adjacent genetic variants tend to be inherited together and can therefore be indirectly captured by marking SNPs [7]. Initially applied primarily in studying complex human diseases, GWASs have gradually been extended to livestock genetic breeding. Currently, numerous marker genes associated with traits such as body weight, the feed conversion ratio, and feed intake have been identified in domestic chickens [8,9].

In response to the challenges encountered in breeding CMCs for improved growth traits, and to elucidate the genetic basis of these traits, this study performed the whole-genome resequencing of 464 phenotyped samples representing the CMC-A population. We evaluated the diversity and degree of inbreeding in the CMC-A population using the sequencing data and pinpointed candidate genes correlated with growth traits via ROH detection and GWASs, thereby facilitating the improved breeding of the Chengkou mountain chicken.

## 2. Results

### 2.1. Phenotypic Data Analysis

**Figure 1:**
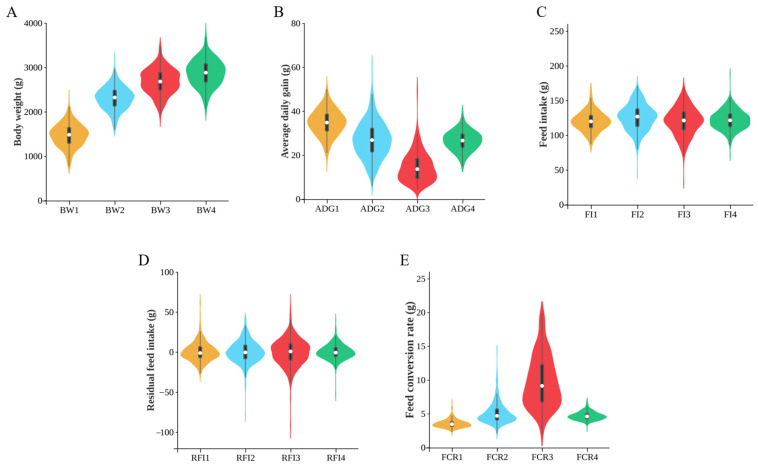
Distribution of the growth traits (n = 464). (**A**) Distribution map of the body weight in different periods (BW1: body weight at 10 weeks; BW2: body weight at 13 weeks; BW3: body weight at 15 weeks; BW4: body weight at 17 weeks). (**B**) Distribution map of the average daily gain in different periods (ADG1: average daily gain at 10 to 13 weeks of age; ADG2: average daily gain at 13 to 15 weeks of age; ADG3: average daily gain at 15 to 17 weeks of age; ADG4: average daily gain at 10 to 17 weeks of age). (**C**) Distribution map of the feed intake in different periods (FI1: feed intake at 10 to 13 weeks of age; FI2: feed intake at 13 to 15 weeks of age; FI3: feed intake at 15 to 17 weeks of age; FI4: feed intake at 10 to 17 weeks of age). (**D**) Distribution map of the residual feed intake in different periods (RFI1: residual feed intake at 10 to 13 weeks of age; RFI2: residual feed intake at 13 to 15 weeks of age; RFI3: residual feed intake at 15 to 17 weeks of age; RFI4: residual feed intake at 10 to 17 weeks of age). (**E**) Distribution map of the feed conversion rate in different periods (FCR1: feed conversion rate at 10 to 13 weeks of age; FCR2: feed conversion rate at 13 to 15 weeks of age; FCR3: feed conversion rate at 15 to 17 weeks of age; FCR4: feed conversion rate at 10 to 17 weeks of age). The same as below.

**Figure 2:**
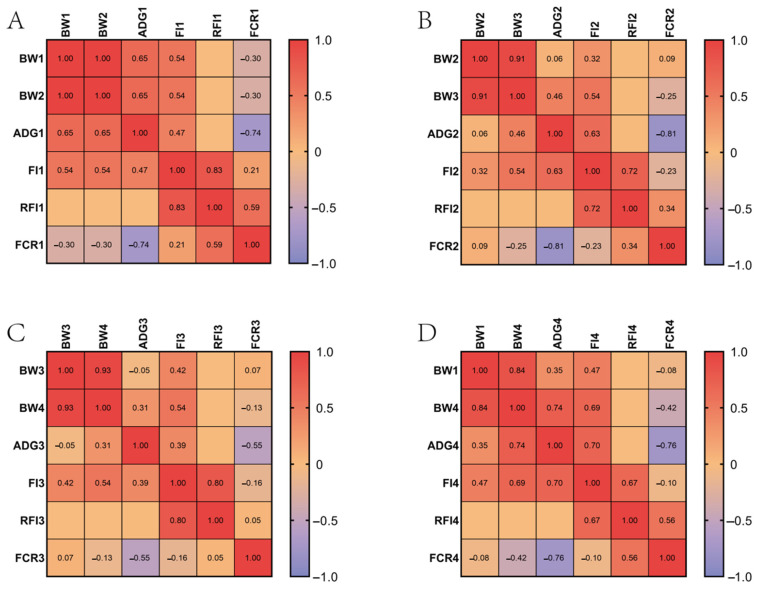
Correlation heat map of growth traits at 10 to 17 weeks of age. (**A**) Correlation heat map of the growth traits at 10 to 13 weeks of age. (**B**) Correlation heat map of the growth traits at 13 to 15 weeks of age. (**C**) Correlation heat map of the growth traits at 15 to 17 weeks of age. (**D**) Correlation heat map of the growth traits at 10 to 17 weeks of age.

To ensure the reliability of the subsequent results, we conducted normality tests, and, except for ADG3 (average daily gain at 15 to 17 weeks of age), all of the traits followed a normal distribution (Figure S1). Figure 1 shows the distribution of the growth traits at 10 to 17 weeks of age. A correlation analysis was performed for the BW (body weight), ADG (average daily gain), FI (feed intake), RFI (residual feed intake), and FCR (feed conversion rate) at each stage. Strong positive correlations were observed among the BW, ADG, and FI at all stages; however, the FCR showed a strong negative correlation with the ADG. The RFI exhibited positive correlations with the FI and FCR but showed no significant correlation with the BW and ADG at the four stages (Figure 2).

### 2.2. Genotypic Data

**Figure 3:**
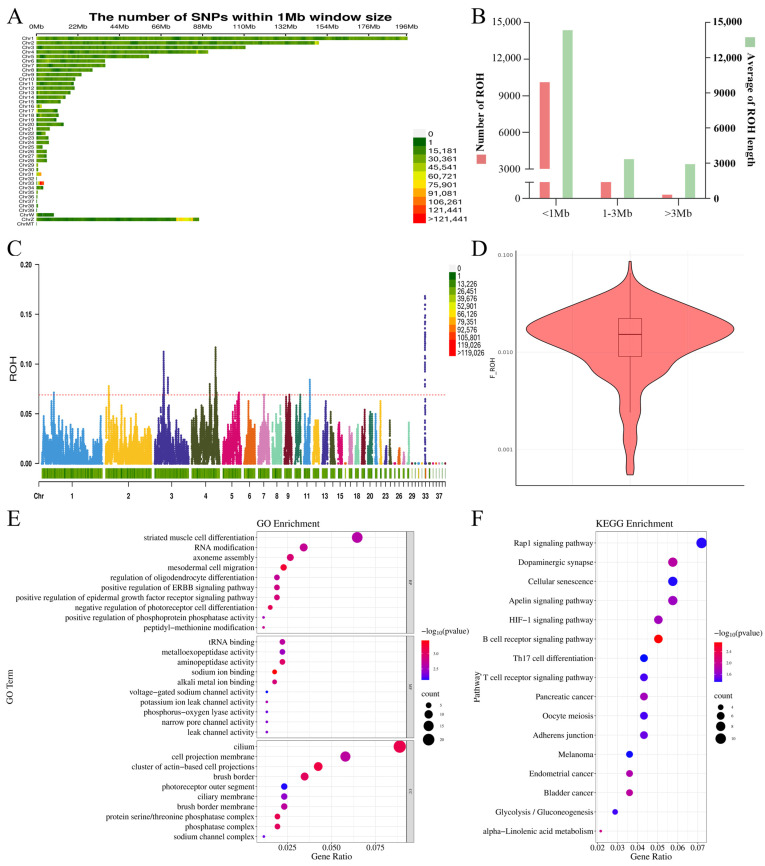
Genome sequencing and ROH island analysis results. (**A**) SNP density distribution on the chromosomes. (**B**) The number of ROHs of different lengths and the average length. (**C**) Manhattan plot of the occurrences of an SNP in ROHs across the population (the red dotted line means the threshold). (**D**) Violin diagram of the inbreeding coefficient FROH based on the ROH. (**E**) GO terms of the candidate gene from the ROH islands, where the most significant ten items in the BP, CC, and MF ontologies were selected to map each ontology. (**F**) KEGG terms of the candidate genes from the ROH islands.

The samples from the 464 CMC-A individuals were sequenced, generating 7901.92 GB of raw data. After quality control, 7897.28 GB of clean data were obtained, with an average of 17.02 GB per individual. The proportion of clean data reached 99.96%, with an average sequencing depth of 16× (Table S1). The population structure was assessed using the first four principal components via principal component analysis (Figure S2). The results revealed that, while most of the individuals clustered together, some exhibited dispersion around the periphery, indicating no obvious stratification. Upon aligning the sequencing results to the reference genome (GRCg7b), 22,204,843 SNP loci were obtained, and using PLINK (v1.9) software to filter for quality control, a total of 3,335,613 high-quality SNP loci were obtained. The distribution of SNPs corresponded to the chromosome lengths (Figure 3A).

### 2.3. Genetic Diversity Analysis and Inbreeding Estimation

The genetic diversity analysis of the CMC-A population revealed average PN, PIC, He, and Ho values of 0.9991, 0.3258, 0.3258, and 0.3207, respectively, showing close proximity. The mean MAF was 0.2386 (Table 1).

Following the ROH detection in the 464 individuals of the CMC-A population, 11,394 ROHs were detected, with an average length of 708.71 Mb (Table 2). The ROHs were primarily distributed on chromosomes 1, 2, 3, 4, and 41, with chromosome 1 exhibiting the highest frequency of detected ROH regions (12.67%). The ROHs were classified based on their lengths: <1 Mb (short), 1~3 Mb (medium), and >3 Mb (long), and the distribution of the ROHs of different lengths was calculated. The mean lengths of the ROHs per individual were calculated, and the results were averaged (Table 3; Figure 3B). In each individual chicken, the <1 MB ROH was the most common, and its total length was longer than the total length of the ROHs of other sizes. The genomic inbreeding coefficient (FROH) was calculated based on the ROH results to assess the degree of inbreeding in the CMC-A population. The FROH for the CMC-A population was 0.017, ranging between 0.0006 and 0.0862 (Figure 3D).

### 2.4. Identification of ROH Island Candidate Genes

The high frequency ROH-related genes (top 0.5%) were used to determine the potential genes under selection pressure in the CMC population (Figure 3C). A total of 414 ROH islands and 317 candidate genes were detected (Table S2), with numerous ROH islands occurring on chromosomes 3, 4, 11, and 33. Among the identified candidate genes, 81 were associated with stress resistance, skeletal growth, muscle development, the fat metabolism, and the energy metabolism (Table 4). These ROH-island-associated genes may be breed-specific, aiding in the identification of the CMC-A population as a broiler breed.

The functional annotation of the identified genes revealed a significant enrichment of 211 GO terms and 16 KEGG pathways (Figure 3E,F). The candidate genes were enriched in the GO annotations, such as the positive regulation of B cell proliferation, the negative regulation of muscle cell differentiation, the myofibril assembly, the regulation of bone remodeling, and the regulation of B cell proliferation (*p* < 0.05). This indicated significant associations with the immune, muscular, and skeletal functions. Additionally, the KEGG analysis identified pathways such as the B cell receptor signaling pathway and T cell receptor signaling pathway, which are associated with the T and B cells.

### 2.5. Whole-Genome Association Study and Identification of Candidate Genes

**Figure 4:**
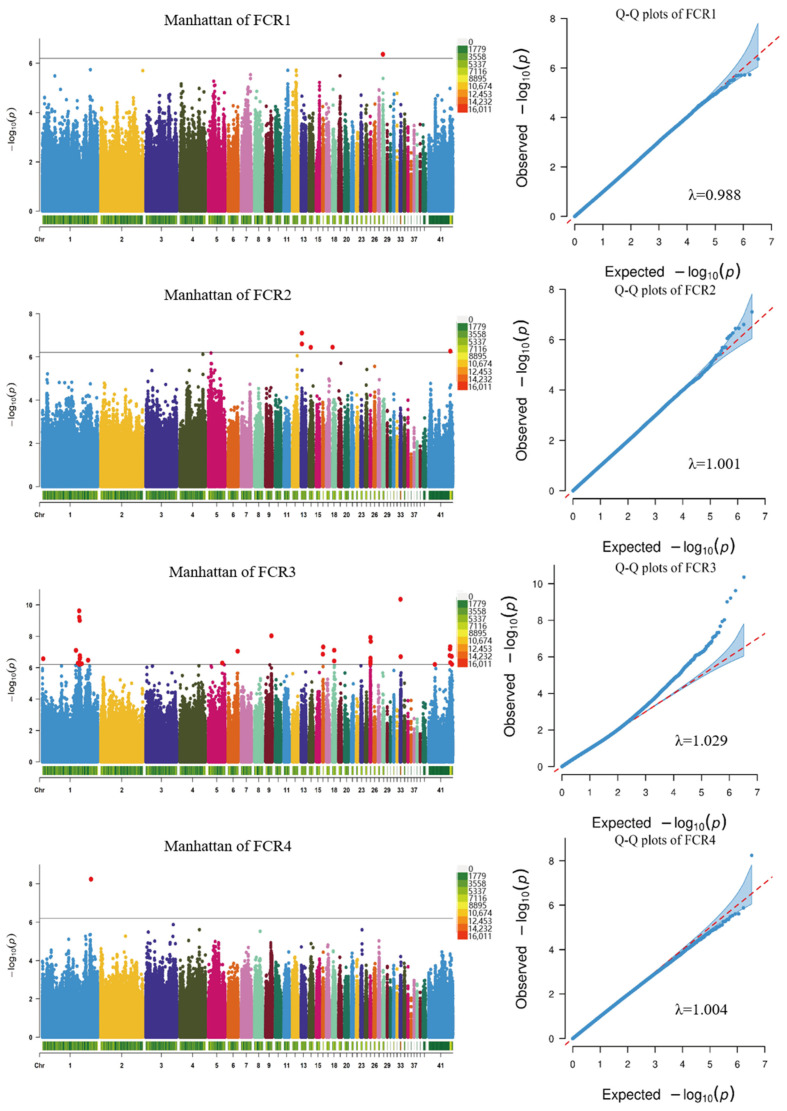
Manhattan chart and QQ plots of the feed conversion ratio GWAS in different periods (The black line in the Manhattan chart indicates the threshold, the same as below).

**Figure 5:**
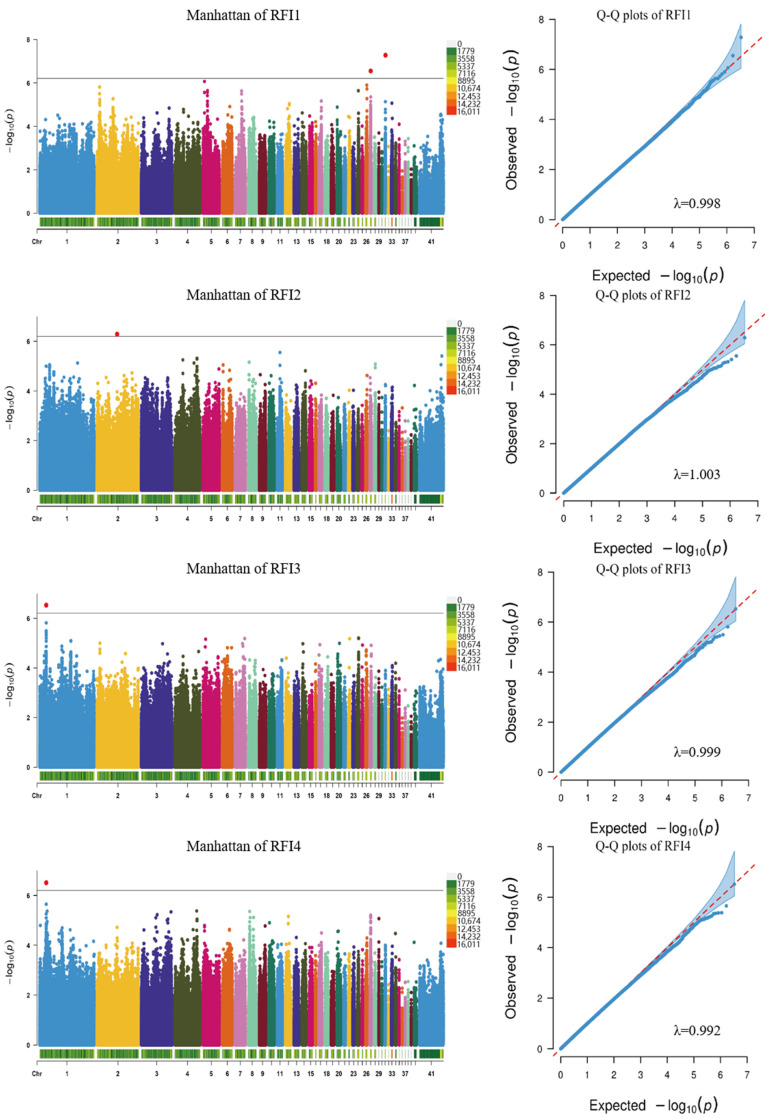
Manhattan chart and QQ plots of the residual feed intake GWAS in different periods.

**Figure 6:**
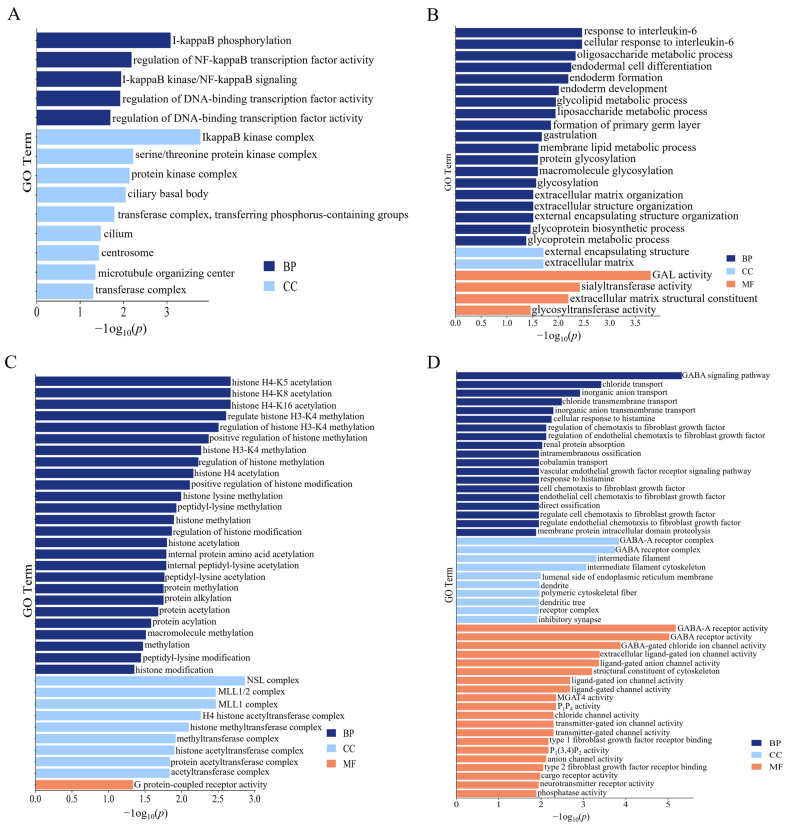
GO enrichment results of the candidate genes selected by the GWAS study. (**A**) GO terms of the candidate genes in the BW (BP and CC). (**B**) GO terms of the candidate genes in the ADG (BP, CC, and MF). (**C**) GO terms of the candidate genes in the RFI (BP, CC, and MF). (**D**) GO terms of the candidate genes in the FCR (BP, CC, and MF).

After sequencing the data filtration, a GWAS analysis was performed on the growth trait phenotypes aged between 10 and 17 weeks using a mixed linear model. The GWAS analysis detected 52 SNPs that were significantly associated with growth traits (Figure 4, Figure 5 and Figures S3–S5), and 71 candidate genes corresponding to SNPs were identified after comparison with reference genomes (Table 5). For the BW, two significant SNP loci were identified on chromosome 1, with BW1 and BW2 identifying the same SNP loci and annotated on the candidate gene ERC1. The ADG was associated with two SNP loci on chromosome 1 and was annotated on the following three candidate genes: *ST3GAL6*, *COL8A1*, and ENSGALG00010006408. The GO analysis showed that the ADG candidate genes were enriched in the glycolipid and liposaccharide metabolic process terms related to sugar and the lipid metabolism, respectively (Figure 6B). The FCR exhibited the most significant loci, totaling 44, with SNP 172549299 on chromosome 1 overlapping with a significant locus for the ADG (Table 5). These SNPs were mainly distributed on chromosomes 1, 25, and 41, with 44 candidate genes annotated. Notably, significant SNPs on chromosome 41 were not annotated on candidate genes, possibly due to its status as a microchromosome. The RFI was associated with five significant loci on chromosomes 1, 2, 27, and 31, and was annotated on the following seven candidate genes: *KANSL1*, *CHIR3B8*, *FAM3C,* ENSGALG00010005410, ENSGALG00010000898, ENSGALG00010000899, and ENSGALG00010001979. According to the GO analysis, the RFI was enriched in 36 terms, mostly related to protein acetylation (Figure 6C).

## 3. Discussion

### 3.1. Phenotypic Analysis

As a significant economic resource in areas such as Chongqing in China, efficient growth traits have always been a crucial selection target in breeding CMCs. The average weight of CMC-A chickens at 17 weeks is 2.8 kg, which is significantly higher compared to the same period of the fifth generation, and has caught up with other locally bred dual-purpose chicken breeds in China after long-term selection (Huiyang Bearded, 2.23 kg; Beijing You chicken, 2.05 kg; Baicheng You chicken, 2.40 kg) [10]. These results indicate the successful breeding efforts of CMCs, resulting in a significantly improved growth performance. Compared to the other stages, the rate of weight gain slows down and the feed conversion ratio decreases from 15 to 17 weeks, suggesting that, to effectively enhance the economic benefits of CMC-A chickens, the feeding period should be shortened and the breeding objectives should focus more on the early growth performance. However, it is worth noting that, at 15–17 weeks, the coefficients of variation for the FCR and ADG are relatively high (Table S3), indicating the insufficient uniformity in the ADG and FCR performance at this stage, which suggests that significant breeding potential still exists.

There exists a strong positive correlation among the BW, ADG, and FI across all stages, while the FCR shows a strong negative correlation with the ADG, which is consistent with the findings by Deng et al. [11]. A lower FCR represents a higher feed conversion efficiency, suggesting that selecting individuals with higher ADGs could enhance the feed conversion efficiency. The RFI showed no significant correlation with the BW or ADG, which is consistent with findings by Yang et al. [12], indicating no phenotypic correlation between the RFI and growth performance.

### 3.2. Genetic Diversity and Inbreeding Analysis

Genetic diversity and the degree of inbreeding within populations are crucial for establishing sustainable breeding programs in poultry. This study employs multiple indices to estimate the genetic diversity and inbreeding relationships within the CMC-A population.

The P_N_ value reflects the proportion of polymorphic loci within a population, while the PIC value indicates the level of population variability [13]. The higher P_N_ and moderate PIC values of the CMC-A population (0.25 < PIC < 0.5) [14] indicate its possession of a considerable proportion of polymorphic loci. The He and Ho, respectively, represent the theoretical and observed heterozygosity at a given locus [15]. In this study, the He and Ho are 0.3258 and 0.3207, respectively. The slightly lower Ho compared to He suggests a higher degree of purity within the CMC-A population, with minimal introduction of exogenous lineages. Overall, the core population of CMC-A roosters exhibits rich genetic diversity.

With the development of whole-genome resequencing and bioinformatics, many methods for inbreeding evaluation using genetic molecular markers have been derived. The FROH is one of them, and the ratio of the total length of the ROH fragment to the total length of the genome is used as the FROH. The inbreeding evaluation based on the FROH has practical utilization value for guiding breeding selection, reducing inbreeding decline, and protecting livestock and poultry genetic resources. ROH patterns vary among different breeds due to factors such as natural geographical environments and breeding strategies. In comparison to other Chinese local breeds, commercial egg layers, and broilers, the average number of ROHs in this study (118) is considerably lower [16]. The quantities, total lengths, and average lengths of ROHs of different lengths in this study show inconsistencies with the findings of Talebi et al. [16]. Long ROHs arise from inbreeding between populations, and the average F_ROH_ in our study population is smaller than that of the samples in Talebi et al.’s study, indicating a lower level of inbreeding in the CMC-A population. Consequently, fewer ROHs are produced. While Bortoluzzi et al. reported contrasting results regarding Dutch local chicken ROHs, it is worth noting that their study was based on SNP chip detection, which tends to overestimate the total length of medium and long ROHs [17,18].

Compared to the inbreeding coefficients calculated based on pedigree records from breeding farms, the F_ROH_ provides a more accurate reflection of the actual inbreeding levels. The average F_ROH_ for the CMC-A population in this study is 0.0863, which is relatively low compared to other native Chinese and foreign breeds [19]. This finding indicates the excellent conservation status of the CMC-A population and the implementation of appropriate conservation and breeding strategies.

### 3.3. Candidate Genes Within ROH Islands

Over the years of artificial selection, CMCs have been bred into distinct lines for meat and egg production. The A line used in this study has been selectively bred for its superior growth performance. ROH islands typically arise from natural or artificial selection and are often closely associated with selected traits. Among the candidate genes identified in this study, a plethora were found to be linked to meat growth traits. In our previous research, we observed the significant upregulation of *SLC25A12* at critical stages of embryonic muscle development in CMCs, and, in this study, the *SLC25A12* gene was identified within ROH islands, suggesting its potential key role in muscle development in CMCs [1]. Additionally, it has been reported that *PDLIM5* positively regulates the proliferation and differentiation of chicken skeletal muscle satellite cells via the p38-MAPK pathway [20]. Fat deposition influences the quality and flavor of chicken meat [21]. The genes identified in this study, such as *PPP1CB*, *MTTP*, and *CERS6*, are associated with the lipid metabolism [22,23,24], providing insights into the genetic mechanisms underlying the superior flavor of the Chengkou mountain chicken.

Indigenous chicken breeds in China are known for their robust resilience [25]. CMCs inhabit Chongqing County, which is characterized by harsh natural environments in mountainous areas with a long history of mountain farming. Through long-term natural selection and breeding, the CMC has developed strong resistance and tolerance to adverse conditions. This study identified 20 genes associated with antioxidant stress and immunity, with some of the genes enriching pathways and terms related to T cells and B cells. T cells and B cells play crucial roles in antigen presentation, antibody production, and cytokine secretion, which are key processes in the immune system, where self-immunity plays a critical role [26,27]. These genes may be linked to the strong resilience of CMCs.

### 3.4. Genome-Wide Association Analysis

Since the first GWAS study on poultry growth traits reported SNPs associated with the abdominal fat percentage in male broilers in 2007 [28], research on trait correlations has been ongoing. Despite being a nationally important genetic resource, research on the genetic architecture of growth traits in the Chengkou mountain chicken is lacking. In this study, a genome-wide association analysis was conducted on the growth traits of 464 CMC-A individuals aged 10–17 weeks, leading to the identification of key candidate genes.

Body weight is one of the most economically important traits in chicken production and breeding, characterized by a complex genetic architecture involving multiple genes and variable inheritance structures [29]. The *ERC1* gene identified in this study encodes a protein involved in regulating neurotransmitter release as a member of the active zone protein RIM-binding protein family [30]. Although there is currently no research on *ERC1* in relation to chicken body weight, it has been found to be significantly associated with the bone mineral content in males in a GWAS study of the skeletal growth of children of European ancestry [31]. In this study, *ERC1* was found to be significantly associated with body weight in both the BW1 and BW2 stages, suggesting its potential role in regulating skeletal growth and, consequently, influencing chicken body weight. In BW3, a significant locus was detected at position 1:169293230 on chromosome 1, which is associated with an Ensembl gene (ENSGALG00010004476) of unknown biological significance. This SNP is located within a QTL region that is significantly associated with chicken body weight on chromosome 1, the 167–179 Mb region, which has been implicated in the QTL associated with chicken body weight [32,33,34].

In addition to the widely used FCR, the RFI, defined as the difference between the actual and expected feed intake, has garnered increasing attention [35]. In this GWAS study, the *KANSL1* and *CHIR3B8* genes were found to be significantly associated with the RFI. Developmental dysplasia of the hip is a common cause of adult hip joint problems, with Xu et al. identifying a missense variant in *KANSL1*, which was associated with a reduced chondrocyte number and diminished cartilage matrix in mice, suggesting it as a novel pathogenic gene for hip dysplasia [36]. Additionally, *KANSL1* has been implicated in cellular processes such as cell proliferation and mitosis [37]. In poultry-related research, Li et al. identified *KANSL1*, *FNDC3A*, and *MPP6* as important candidate genes influencing visceral organ development in a GWAS study on chicken visceral weights [38]. *CHIR3B8* is the chicken homologue of immunoglobulin-like receptors [39]. A study on the *CHIR* genes in different chicken breeds found a significant over-representation of *CHIR* genes in broilers compared to red junglefowl and layers. The difference in the *CHIR* gene numbers between broilers and layers suggests that different breeding directions may have led to variations in the *CHIR* genes, implying that *CHIR* genes may have evolved in response to selective breeding needs [40]. *CHIR* could potentially be a key gene in the selection for growth traits.

The FCR, as a crucial indicator for assessing the feed efficiency in chickens, has long been a pivotal trait in breeding, with numerous studies delving into its underlying genetic architecture. In this GWAS study, 44 SNP loci and a corresponding 18 candidate genes associated with the FCR were identified. Neurons in the Lateral Hypothalamic Area (LHA) regulate fat tissue, modulating the food intake and energy balance. *GABRA5*-positive neurons in the LHA, inhibited by gamma-aminobutyric acid (GABA) released by astrocytes, led to increased fat thermogenesis and significantly reduced weight gain in obese mice upon the silencing of GABA synthesis [41]. In this study, the association of the *GABRA5* gene with the FCR in Chengkou mountain chickens was identified, suggesting that *GABRA5* may be related to the fat heat production of Chengkou mountain chickens, thus affecting the fat accumulation and FCR. Carmelo et al. compared the RNA-seq data from two pig breeds with different feed conversion rates and identified 13 differentially expressed genes, including *MGAT4A*. The functional enrichment analysis of these differentially expressed genes revealed their association with mitochondrial function [42]. Moreover, *MGAT4A* has been linked to beef tenderness in Hanwoo cattle [43], suggesting a potential connection between *MGAT4A* and the muscle metabolism. *NPAS2* is considered a key gene regulating circadian rhythms in animals, with disrupted circadian rhythms leading to disease development [44]. Normal circadian rhythms are beneficial for shaping the gut microbiota environment in poultry [45], providing higher productivity, better bone health, and reduced stress in chicken flocks [46]. *FGF18* accelerates osteoblast differentiation to promote bone formation and plays a crucial role in skeletal growth and development [47]. Hu et al. revealed that 75 differentially methylated genes, including *FGF18*, may play a critical role in the growth rate of broilers at 7 weeks [48]. *ARHGEF1* plays a significant role in cellular processes such as insulin secretion, insulin signal transduction, and the lipid metabolism, and is associated with type 2 diabetes and insulin resistance [49]. In poultry, elevated insulin levels enhance liver glucose uptake, promote the de novo synthesis of fatty acids, and facilitate fat deposition, thereby accelerating poultry growth [50,51]. Among the numerous candidate genes associated with the FCR detected in this experiment, *GABRA5*, *MGAT4A*, *NPAS2*, *FGF18*, and *ARHGEF11* were identified as five crucial candidate genes. These genes may influence the weight gain of Chengkou mountain chickens, thereby affecting their FCRs through mechanisms involving fat thermogenesis, the muscle metabolism, circadian rhythms, skeletal growth, and the regulation of insulin levels. However, the specific biological regulatory mechanisms underlying these effects warrant further investigation.

## 4. Materials and Methods

### 4.1. Animal Rearing and Phenotype Measuring

All of the experimental and animal handling procedures strictly adhered to the protocol approved by the Southwest University Experimental Animal Ethics Committee. The CMC-A roosters were individually housed in chicken cages at the Chengkou Mountain Chicken Genetic Resources Research Institute, Chongqing, China. Healthy individuals with complete data (n = 464) were randomly selected for genotype profiling, and blood samples were collected from wing veins at 17 weeks of age for sequencing.

The growth traits of CMCs aged 10 to 17 weeks were collected and divided into the following four time points: 10–13, 13–15, 15–17, and 10–17 weeks. Growth traits, including the body weight (BW), average daily gain (ADG), feed intake (FI), residual feed intake (RFI), and feed conversion rate (FCR), were statistically analyzed for each stage using SPSS version 26.0. A correlation analysis of the growth traits in different stages was conducted using GraphPad Prism version 9.5, and heat maps were generated accordingly.

### 4.2. Sequencing and SNP Calling

Genomic DNA was extracted using a Magnetic Universal Genomic DNA Kit, followed by sample sequencing on the DNBSEQ-T7 platform (Complete Genomics and MGI Tech, Shenzhen, China) post-quality control. To eliminate interference from low-quality reads and to ensure the quality of the subsequent analysis, we cleaned the raw data using Fastq (v0.20.0) [52]. The filtering criteria included the removal of adapter contamination, length filtering (discarding reads with a length of ≤50 bp on either end), and quality filtering (removing bases with a quality value of <20) using a sliding window approach with a window size of 2 bp and a step size of 1 bp. This was followed by the trimming of windows with an average quality below 25.

Thereafter, the clean data in fastq format were aligned to the reference genome (GRCg7b; https://www.ncbi.nlm.nih.gov/datasets/genome/GCF_016699485.2/) (accessed on 1 March 2024) using bwa (v0.7.17-r1188) [53], and subsequently sorted using sambamba (v0.8.2). Based on the reference genome, GATK (v4.2.6.1) [54] was used for accurate mutation identification and genotyping.

### 4.3. ROH Island Identification and Significant SNP Identification

Detected SNPs were filtered using the following criteria: an SNP call rate > 90%, a minimum allele frequency > 0.05, and a Hardy–Weinberg equilibrium *p*-value > 10^−6^, where only autosomal SNPs were retained. ROH detection was conducted using PLINK (v1.9) with the following parameters: a sliding window of 50 SNPs, a minimum SNP density of 1/50 kb, an allowance of up to 20% of heterozygous and 5 missing genotypes per individual, a minimum distance of 1 Mb between adjacent ROHs, and a minimum ROH length of 500 kb to mitigate the influence of strong linkage disequilibria between loci. The share of each SNP in the ROHs was calculated as a percentage, with the top 0.5% of SNPs being selected as significant SNPs.

### 4.4. Genetic Diversity Analysis and Inbreeding Level Assessment

Further SNP filtering was performed to eliminate SNPs with a genotype missing rate of >0.1, a minor allele frequency of <5%, and a deviation from the Hardy–Weinberg equilibrium (*p* < 0.0001). Samples with a missing rate of >0.2 and genetic loci with a minor allele frequency (MAF) of <0.01 were excluded. Additionally, samples with heterozygosity rates deviating ±3 SD from the mean and the high linkage disequilibrium SNP loci (LD < 0.5) were excluded.

Genetic diversity was evaluated using the expected heterozygosity (H_E_), observed heterozygosity (H_O_), percentage of polymorphic loci (P_N_), minor allele frequency (MAF), and polymorphic information content (PIC). The H_E_, H_O_, P_N_, PIC, and MAF were calculated using PLINK (v1.9). The inbreeding coefficient (F_ROH_) was assessed based on the ROH identification results and calculated as follows:

(1) $$F_{ROH}=\frac{\Sigma L_{ROH}}{L_{genome}}\;,$$

where ∑*L*_*ROH*_ is the total length of the ROH segments on the autosomes and *L*_*genome*_ is the total physical length of the autosomes.

### 4.5. GWAS

The SNP loci intended for the genetic diversity calculation were re-filtered using the same criteria unchanged, except for the inclusion of LD < 0.7, to remove the linkage disequilibrium. A mixed linear model [55] was employed for the GWAS study, as follows:

(2) $$y=X\beta +Z_{k}\gamma_{k}+\xi +e$$

In the model, y represents the phenotype, Xβ represents the population structure effects and fixed effects, and Z_k_γ_k_ represents the effects of the markers to be tested; ξ~N (0, Kφ2) denotes the polygenic effects, where K is the kinship matrix inferred from the markers, and e~N (0, Iσ2) represents the residual effects. The GAPIT3 (V3.4) tool was used to run the model, calculating the *p*-values for each SNP locus. The genome-wide significance threshold was determined as 6.16 × 10^−7^ (=1/1,622,570) after correction using the simpleM algorithm [56].

### 4.6. Gene Ontology (GO) and Kyoto Encyclopedia of Genes and Genomes (KEGG) Enrichment of Candidate Genes

Candidate genes were identified based on their physical position and function based on the GRCg7b reference genome. The significant SNPs identified through the GWAS study and ROH islands were annotated to their corresponding genes using ANNOVAR software (20191024). Subsequently, genes located in intergenic regions were extracted, and GO enrichment and KEGG pathway analyses were performed on the OmicShare platform (https://www.omicshare.com/tools/) (accessed on 21 August 2024).

## 5. Conclusions

In this study, genetic diversity analysis and growth trait identification of CMC-A roosters were performed based on whole-genome sequencing. The results showed that the CMC-A roosters had a high genetic diversity and a low inbreeding level, indicating the effectiveness of the current conservation and breeding efforts. In addition, 52 SNP loci and 71 candidate genes related to growth traits were detected by the ROH islands and GWAS analysis, which may have an impact on the stress resistance, muscle growth, and fat deposition of the CMCs. These findings are helpful in further understanding the genetic structure of the growth traits of Chengkou mountain chickens and provide a theoretical basis for subsequent breeding work.

## Figures and Tables

**Table 1:** Population diversity of the Chengkou mountain chicken.

MAF	He	Ho	PIC	Ho
0.238584	0.32583	0.320666	0.325829	0.320666

**Table 2:** ROH distribution and genome inbreeding coefficient of the Chengkou mountain chicken.

	Max	Min	Aver	Std
ROH length (Kb)	3297.98	500.00	708.71	223.51
Total length of individual ROHs (Kb)	89,803.00	570.58	17,403.19	11,183.42
Number of ROHs	118.00	1.00	24.56	15.24
FROH	0.086274	0.000548	0.017	0.011

**Table 3:** Distribution and average length of ROHs of different lengths.

	Length	Number	Max	Min	Aver	Std
<1 Mb	6,739,650	10,319	999.95	500.004	14,556.48	9059.392
1–3 Mb	1,322,810	1071	2693.12	1000.601	3584.85	2847.158
>3 Mb	12,620	4	3297.98	3017.774	3155.02	153.687

**Table 4:** Functions of some candidate genes in the ROH islands.

Function of Some Candidate Genes	Gene Names
Stress resistance	*CD38*, *BTBD9*, *ADH6*, *CISD2*, *BDH2*, *SLC39A8*, *NFKB1*, *TLR3*, *FAM149A*, *BANK1*, *GRPEL1*, *SORCS2*, *PFKP*, *ACOX3*, *HSPA2*, *LRFN2*, *TMEM179*, *SIVA1*, *INF2*, *FASTKD1*
Muscle development	*CPEB2*, *NCAPG*, *LAP3*, *LDB2*, *ZNF318*, *QKI*, *PDLIM5*, *NEUROG2*, *PITX2*, *WDR37*, *MYF6*, *MYF5*, *SYNE2*, *PPP2R5E*, *BRE*, *AKT1*, *JAG2*, *PDK1*, *METTL8*, *SLC25A12*
Bone growth	*NKX3-2*, *CYTL1*, *SLC35B2*, *FAM98A*, *CAPN11*, *DLK2*, *SNORD71*, *ALPK1*, *EMCN*, *PPP3CA*, *DIP2C*, *RPS6KA2*, *CPZ*, *GPHB5*, *DLX2*, *GALNT3*
Energy metabolism	*HS3ST1*, *NSG1*, *GLO1*, *SLC29A1*, *PHLPP2*, *ELOVL6*, *EGF*, *PITRM1*, *FOSL2*, *G6PC2*, *CSRNP3*
Fat deposition	*FBXL5*, *RASGRP3*, *JAKMIP1*, *COG8*, *PLA2G12A*, *MTTP*, *KLF6*, *ACSS3*, *RHOJ*, *PPP1CB*, *GPR132*, *PPIG*, *LRP2*, *CERS6*

**Table 5:** SNPs and genes associated with growth traits.

Chr	SNP ID	Ref/Alt	Func.refGene	Ensembl ID	Gene_Name	Phenotypes
1	1:60821062	A/G	intronic	ENSGALG00010012399	*ERC1*	BW1, BW2
1	1:169293230	T/C	ncRNA_intronic	ENSGALG00010004476	NA	BW3
1	1:84523514	G/A	intronic	ENSGALG00010005423; ENSGALG00010005474	*ST3GAL6*; *COL8A1*	ADG2
1	1:172549299	T/G	intergenic	ENSGALG00010006408; ENSGALG00010007373	NA;NA	ADG5, FCR5
28	28:992707	A/G	intronic	ENSGALG00010028357	*SPPL2C*	FCR1
13	13:2852686	T/C	intronic	ENSGALG00010010399	*FGF18*	FCR2
13	13:2856342	G/A	intronic	ENSGALG00010010399	*FGF18*	FCR2
14	14:6259710	G/A	intronic	ENSGALG00010017898	*NHERF2*	FCR2
18	18:250974	T/C	intergenic	NONE; ENSGALG00010030050	NA; NA	FCR2
41	41:76970673	T/C	intergenic	NONE;NONE	NA; NA	FCR2
1	1:2782842	C/T	exonic	ENSGALG00010007596	NA	FCR3
1	1:119168544	A/G	intergenic	ENSGALG00010003392; ENSGALG00010003275	NA; *MBTPS2*	FCR3
1	1:126186942	C/T	intergenic	ENSGALG00010001468; ENSGALG00010003235	*ANOS1*; gga-mir-7448	FCR3
1	1:130412664	T/C	UTR3	ENSGALG00010002753	*SLC9A7*	FCR3
1	1:130442457	A/G	intergenic	ENSGALG00010003157; ENSGALG00010002800	NA; *TUBGCP5*	FCR3
1	1:130442500	A/G	intergenic	ENSGALG00010003157; ENSGALG00010002800	NA; *TUBGCP5*	FCR3
1	1:130619948	G/T	intronic	ENSGALG00010002886	*HERC2*	FCR3
1	1:131241497	A/G	intronic	ENSGALG00010002907	*GABRG3*	FCR3
1	1:131389633	A/G	intronic	ENSGALG00010002927; ENSGALG00010002942	*GABRA5*; *GABRB3*	FCR3
1	1:132406216	C/A	upstream	ENSGALG00010015669	*INPP4A*	FCR3
1	1:132427487	C/T	intronic	ENSGALG00010015716	*MGAT4A*	FCR3
1	1:133078359	T/C	intronic	ENSGALG00010015886	*AFF3*	FCR3
1	1:133453381	T/A	intronic	ENSGALG00010013436	*NPAS2*	FCR3
1	1:139215439	G/T	intergenic	ENSGALG00010014379; ENSGALG00010014396	*CARS2*; *ING1*	FCR3
1	1:162102310	A/G	intronic	ENSGALG00010002381	*TDRD3*	FCR3
5	5:49414192	C/T	exonic	ENSGALG00010015137	*AMN*	FCR3
6	6:34082468	G/A	intronic	ENSGALG00010002712	*PTPRE*	FCR3
9	9:20447352	G/A	ncRNA_intronic	ENSGALG00010001602	NA	FCR3
16	16:291729	C/T	intronic	ENSGALG00010003661	NA	FCR3
16	16:1610013	C/A	intergenic	ENSGALG00010002238; ENSGALG00010002354	NA; NA	FCR3
18	18:6176886	T/G	ncRNA_intronic	ENSGALG00010030069	NA	FCR3
18	18:6204681	G/A	ncRNA_intronic	ENSGALG00010030069	NA	FCR3
25	25:1112852	C/G	intronic	ENSGALG00010027826	*ARHGEF11*	FCR3
25	25:1187425	A/G	exonic	ENSGALG00010027624	NA	FCR3
25	25:1296967	C/T	intronic	ENSGALG00010027728	*CRNN*	FCR3
25	25:1526371	G/A	intronic	ENSGALG00010028545; ENSGALG00010028558	NA; NA	FCR3
25	25:1574011	A/G	intronic	ENSGALG00010028882; ENSGALG00010028886	NA;NA	FCR3
25	25:2434093	C/T	upstream;downstream	ENSGALG00010028878; ENSGALG00010028875	*IGSF9*; *DUSP23*	FCR3
33	33:1553885	G/A	exonic	ENSGALG00010001909	NA	FCR3
33	33:2515964	T/G	exonic	ENSGALG00010002057	NA	FCR3
41	41:22283106	T/C	intergenic	NONE; NONE	NA; NA	FCR3
41	41:74728417	G/T	intergenic	NONE; NONE	NA; NA	FCR3
41	41:76016616	C/T	intergenic	NONE; NONE	NA; NA	FCR3
41	41:76442122	T/C	intergenic	NONE; NONE	NA; NA	FCR3
41	41:77227385	G/A	intergenic	NONE; NONE	NA; NA	FCR3
41	41:81787412	A/G	intergenic	NONE; NONE	NA; NA	FCR3
41	41:82785087	T/C	intergenic	NONE; NONE	NA; NA	FCR3
27	27:2560032	G/A	intronic	ENSGALG00010024809	*KANSL1*	RFI1
31	31:2019899	G/A	intronic	ENSGALG00010005410; ENSGALG00010005529	NA; *CHIR3B8*	RFI1
2	2:72477770	G/C	intergenic	ENSGALG00010000898; ENSGALG00010000899	NA; NA	RFI2
1	1:23290767	G/A	intergenic	ENSGALG00010001979; ENSGALG00010001264	NA; *FAM3C*	RFI3
1	1:23332705	T/G	intergenic	ENSGALG00010001979; ENSGALG00010001264	NA; *FAM3C*	RFI4, RFI5

Chr: SNP chromosome number of the SNP; SNP ID: SNP name; Ref/Alt: reference allele/substitute allele; Func.refGene: functional comment; Ensembl ID: candidate gene name of a gene in the Ensembl database; Gene_name: candidate gene name; Phenotypes: significant SNPs and genetic equivalents; NONE: no Ensembl ID; NA: no gene name.

## Data Availability

All of the relevant data for this study are presented in the manuscript and its Supplementary Files.

## Supplementary Materials

The following supporting information can be downloaded at: https://www.mdpi.com/article/10.3390/ijms252312939/s1.

## References

[1] Ren L., Liu A., Wang Q., Wang H., Dong D., Liu L. (2021). Transcriptome analysis of embryonic muscle development in Chengkou Mountain Chicken. BMC Genom..

[2] Dou D., Shen L., Zhou J., Cao Z., Luan P., Li Y., Xiao F., Guo H., Li H., Zhang H. (2022). Genome-wide association studies for growth traits in broilers. BMC Genom..

[3] Ceballos F.C., Joshi P.K., Clark D.W., Ramsay M., Wilson J.F. (2018). Runs of homozygosity: Windows into population history and trait architecture. Nat. Rev. Genet..

[4] Biscarini F., Nicolazzi E.L., Stella A., Boettcher P.J., Gandini G. (2015). Challenges and opportunities in genetic improvement of local livestock breeds. Front. Genet..

[5] Bosse M., Megens H.J., Madsen O., Crooijmans R.P.M.A., Ryder O.A., Austerlitz F., Groenen M.A.M., Cara M.A.R. (2015). Using genome-wide measures of coancestry to maintain diversity and fitness in endangered and domestic pig populations. Genome Res..

[6] Mastrangelo S., Ciani E., Sardina M.T., Sottile G., Pilla F., Portolano B., Bi. Ov. Ita Consortium (2018). Runs of homozygosity reveal genome-wide autozygosity in Italian sheep breeds. Anim. Genet..

[7] Liao R., Zhang X., Chen Q., Wang Z., Wang Q., Yang C., Pan Y. (2016). Genome-wide association study reveals novel variants for growth and egg traits in Dongxiang blue-shelled and White Leghorn chickens. Anim. Genet..

[8] Tarsani E., Kranis A., Maniatis G., Avendano S., Hager-Theodorides A.L., Kominakis A. (2019). Discovery and characterization of functional modules associated with body weight in broilers. Sci. Rep..

[9] Cao X., Wang Y., Shu D., Qu H., Luo C., Hu X. (2020). Food intake-related genes in chicken determined through combinatorial genome-wide association study and transcriptome analysis. Anim. Genet..

[10] Wang H., Zhao X., Wen J., Wang C., Zhang X., Ren X., Zhang J., Li H., Muhatai G., Qu L. (2023). Comparative population genomics analysis uncovers genomic footprints and genes influencing body weight trait in Chinese indigenous chicken. Poult. Sci..

[11] Deng X.M., Li J.Y., Li N., Wu C.X. (2001). Genetic analysis of important growth trait based on F-2 resource population in chicken. Yi Chuan Xue Bao.

[12] Yang L., Wang X., He T., Xiong F., Chen X., Chen X., Jin S., Geng Z. (2020). Association of residual feed intake with growth performance, carcass traits, meat quality, and blood variables in native chickens. J. Anim. Sci..

[13] Hillel J., Groenen M.A.M., Tixier-Boichard M., Korol A.B., David L., Kirzhner V.M., Burke T., Barre-Dirie A., Crooijmans R.P.M.A., Elo K. (2003). Biodiversity of 52 chicken populations assessed by microsatellite typing of DNA pools. Genet. Sel. Evol..

[14] Azimu W., Manatbay B., Li Y., Kaimaerdan D., Wang H.E., Reheman A., Muhatai G. (2018). Genetic diversity and population structure analysis of eight local chicken breeds of Southern Xinjiang. Br. Poult. Sci..

[15] Kirikci K., Cam M.A., Mercan L. (2020). Genetic diversity and relationship among indigenous Turkish Karayaka sheep subpopulations. Arch. Anim. Breed..

[16] Talebi R., Szmatoła T., Mészáros G., Qanbari S. (2020). Runs of Homozygosity in Modern Chicken Revealed by Sequence Data. G3 Genes Genomes Genet..

[17] Purfield D.C., Berry D.P., McParland S., Bradley D.G. (2012). Runs of homozygosity and population history in cattle. BMC Genet..

[18] Bortoluzzi C., Crooijmans R.P.M.A., Bosse M., Hiemstra S.J., Groenen M.A.M., Megens H.J. (2018). The effects of recent changes in breeding preferences on maintaining traditional Dutch chicken genomic diversity. Heredity.

[19] Dementieva N.V., Kudinov A.A., Larkina T.A., Mitrofanova O.V., Dysin A.P., Terletsky V.P., Tyshchenko V.I., Griffin D.K., Romanov M.N. (2020). Genetic Variability in Local and Imported Germplasm Chicken Populations as Revealed by Analyzing Runs of Homozygosity. Animals.

[20] He H., Yin H., Yu X., Zhang Y., Ma M., Li D., Zhu Q. (2021). PDLIM5 Affects Chicken Skeletal Muscle Satellite Cell Proliferation and Differentiation via the p38-MAPK Pathway. Animals.

[21] Tian S., Tang W., Zhong Z., Wang Z., Xie X., Liu H., Chen F., Liu J., Han Y., Qin Y. (2023). Identification of Runs of Homozygosity Islands and Functional Variants in Wenchang Chicken. Animals.

[22] Hammerschmidt P., Ostkotte D., Nolte H., Gerl M.J., Jais A., Brunner H.L., Sprenger H.G., Awazawa M., Nicholls H.T., Turpin-Nolan S.M. (2019). CerS6-Derived Sphingolipids Interact with Mff and Promote Mitochondrial Fragmentation in Obesity. Cell.

[23] Peng H., Chiu T.Y., Liang Y.J., Lee C.J., Liu C.S., Suen C.S., Yen J.J.Y., Chen H.T., Hwang M.J., Hussain M.M. (2021). PRAP1 is a novel lipid-binding protein that promotes lipid absorption by facilitating MTTP-mediated lipid transport. J. Biol. Chem..

[24] Kim J., Ahn D., Chung S.J. (2022). Chebulinic Acid Suppresses Adipogenesis in 3T3-L1 Preadipocytes by Inhibiting PPP1CB Activity. Int. J. Mol. Sci..

[25] Weng Z., Xu Y., Li W., Chen J., Zhong M., Zhong F., Du B., Zhang B., Huang X. (2020). Genomic variations and signatures of selection in Wuhua yellow chicken. PLoS ONE.

[26] Kumar B.V., Connors T.J., Farber D.L. (2018). Human T Cell Development, Localization, and Function throughout Life. Immunity.

[27] Raza I.G.A., Clarke A.J. (2021). B Cell Metabolism and Autophagy in Autoimmunity. Front. Immunol..

[28] Abasht B., Lamont S.J. (2007). Genome-wide association analysis reveals cryptic alleles as an important factor in heterosis for fatness in chicken F2 population. Anim. Genet..

[29] Yuan Y., Peng D., Gu X., Gong Y., Sheng Z., Hu X. (2018). Polygenic Basis and Variable Genetic Architectures Contribute to the Complex Nature of Body Weight —A Genome-Wide Study in Four Chinese Indigenous Chicken Breeds. Front. Genet..

[30] Held R.G., Kaeser P.S. (2018). ELKS active zone proteins as multitasking scaffolds for secretion. Open Biol..

[31] Mitchell J.A., Chesi A., Elci O., McCormack S.E., Roy S.M., Kalkwarf H.J., Lappe J.M., Gilsanz V., Oberfield S.E., Shepherd J.A. (2016). Physical Activity Benefits the Skeleton of Children Genetically Predisposed to Lower Bone Density in Adulthood. J. Bone Miner. Res..

[32] Liu X., Li H., Wang S., Hu X., Gao Y., Wang Q., Li N., Wang Y., Zhang H. (2007). Mapping quantitative trait loci affecting body weight and abdominal fat weight on chicken chromosome one. Poult. Sci..

[33] Besnier F., Wahlberg P., Rönnegård L., Ek W., Andersson L., Siegel P.B., Carlborg O. (2011). Fine mapping and replication of QTL in outbred chicken advanced intercross lines. Genet. Sel. Evol..

[34] Xie L., Luo C., Zhang C., Zhang R., Tang J., Nie Q., Ma L., Hu X., Li N., Da Y. (2012). Genome-Wide Association Study Identified a Narrow Chromosome 1 Region Associated with Chicken Growth Traits. PLoS ONE.

[35] Romero L.F., Zuidhof M.J., Renema R.A., Robinson F.E., Naeima A. (2009). Nonlinear mixed models to study metabolizable energy utilization in broiler breeder hens. Poult. Sci..

[36] Xu X., Bi X., Wang J., Gui R., Li T., Li L., Wang B. (2022). Identification of KANSL1 as a novel pathogenic gene for developmental dysplasia of the hip. J. Mol. Med..

[37] Meunier S., Shvedunova M., Van Nguyen N., Avila L., Vernos I., Akhtar A. (2015). An epigenetic regulator emerges as microtubule minus-end binding and stabilizing factor in mitosis. Nat. Commun..

[38] Li Y., Liu X., Bai X., Wang Y., Leng L., Zhang H., Li Y., Cao Z., Luan P., Xiao F. (2022). Genetic parameters estimation and genome-wide association studies for internal organ traits in an F2 chicken population. J. Anim. Breed. Genet..

[39] Dennis G., Kubagawa H., Cooper M.D. (2000). Paired Ig-like receptor homologs in birds and mammals share a common ancestor with mammalian Fc receptors. Proc. Natl. Acad. Sci. USA.

[40] Sparling B.A., Ng T.T., Carlo-Allende A., McCarthy F.M., Taylor R.L., Drechsler Y. (2024). Immunoglobulin-like receptors in chickens: Identification, functional characterization, and renaming to cluster homolog of immunoglobulin-like receptors. Poult. Sci..

[41] Sa M., Yoo E.S., Koh W., Park M.G., Jang H.J., Yang Y.R., Bhalla M., Lee J.H., Lim J., Won W. (2023). Hypothalamic GABRA5-positive neurons control obesity via astrocytic GABA. Nat. Metab..

[42] Carmelo V.A.O., Kadarmideen H.N. (2020). Genome Regulation and Gene Interaction Networks Inferred from Muscle Transcriptome Underlying Feed Efficiency in Pigs. Front. Genet..

[43] Chung Y., Jang S.S., Kang D.H., Kim Y.K., Kim H.J., Chung K.Y., Choi I., Lee S.H. (2023). Identification of potential biomarkers associated with meat tenderness in Hanwoo (Korean cattle): An expression quantitative trait loci analysis. Anim. Genet..

[44] Peng L.U., Bai G., Pang Y. (2021). Roles of NPAS2 in circadian rhythm and disease. Acta Biochim. Biophys. Sin..

[45] Hieke A.-S.C., Hubert S.M., Athrey G. (2019). Circadian disruption and divergent microbiota acquisition under extended photoperiod regimens in chicken. PeerJ.

[46] Jiang S., Fu Y., Cheng H. (2023). Daylight exposure and circadian clocks in broilers: Part I—Photoperiod effect on broiler behavior, skeletal health, and fear response. Poult. Sci..

[47] Nagayama T., Okuhara S., Ota M.S., Tachikawa N., Kasugai S., Iseki S. (2013). FGF18 accelerates osteoblast differentiation by upregulating Bmp2 expression. Congenit. Anom..

[48] Hu Y., Xu H., Li Z., Zheng X., Jia X., Nie Q., Zhang X. (2013). Comparison of the genome-wide DNA methylation profiles between fast-growing and slow-growing broilers. PLoS ONE.

[49] Liu J., Chen X., Guo Q., Ma X., Zhang J., Huang X., Zhang X., Zhang S. (2011). Association of ARHGEF11 R1467H polymorphism with risk for type 2 diabetes mellitus and insulin resistance in Chinese population. Mol. Biol. Rep..

[50] Tarlow D.M., Watkins P.A., Reed R.E., Miller R.S., Zwergel E.E., Lane M.D. (1977). Lipogenesis and the synthesis and secretion of very low density lipoprotein by avian liver cells in nonproliferating monolayer culture. Hormonal effects. J. Cell Biol..

[51] Hermier D. (1997). Lipoprotein metabolism and fattening in poultry. J. Nutr..

[52] Chen S., Zhou Y., Chen Y., Gu J. (2018). fastp: An ultra-fast all-in-one FASTQ preprocessor. Bioinformatics.

[53] Li H., Durbin R. (2010). Fast and accurate long-read alignment with Burrows-Wheeler transform. Bioinformatics.

[54] Poplin R., Ruano-Rubio V., Depristo M.A., Fennell T.J., Banks E. (2017). Scaling accurate genetic variant discovery to tens of thousands of samples. bioRxiv.

[55] Yu J., Pressoir G., Briggs W.H., Vroh B.I., Yamasaki M., Doebley J.F., McMullen M.D., Gaut B.S., Nielsen D.M., Holland J.B. (2006). A unified mixed-model method for association mapping that accounts for multiple levels of relatedness. Nat. Genet..

[56] Gao X., Starmer J., Martin E.R. (2008). A multiple testing correction method for genetic association studies using correlated single nucleotide polymorphisms. Genet. Epidemiol..

