# Supplementary material for: Genetic Diversity Analysis and Identification of Candidate Genes for Growth Traits in Chengkou Mountain Chicken
Source: Int J Mol Sci. 2024 Dec 2;25(23):12939. doi: 10.3390/ijms252312939 (PMC11641596; doi:10.3390/ijms252312939)
Supplement: Supplementary file 1 [file ijms-25-12939-s001.zip › Supplementary Figure and Table.pdf]

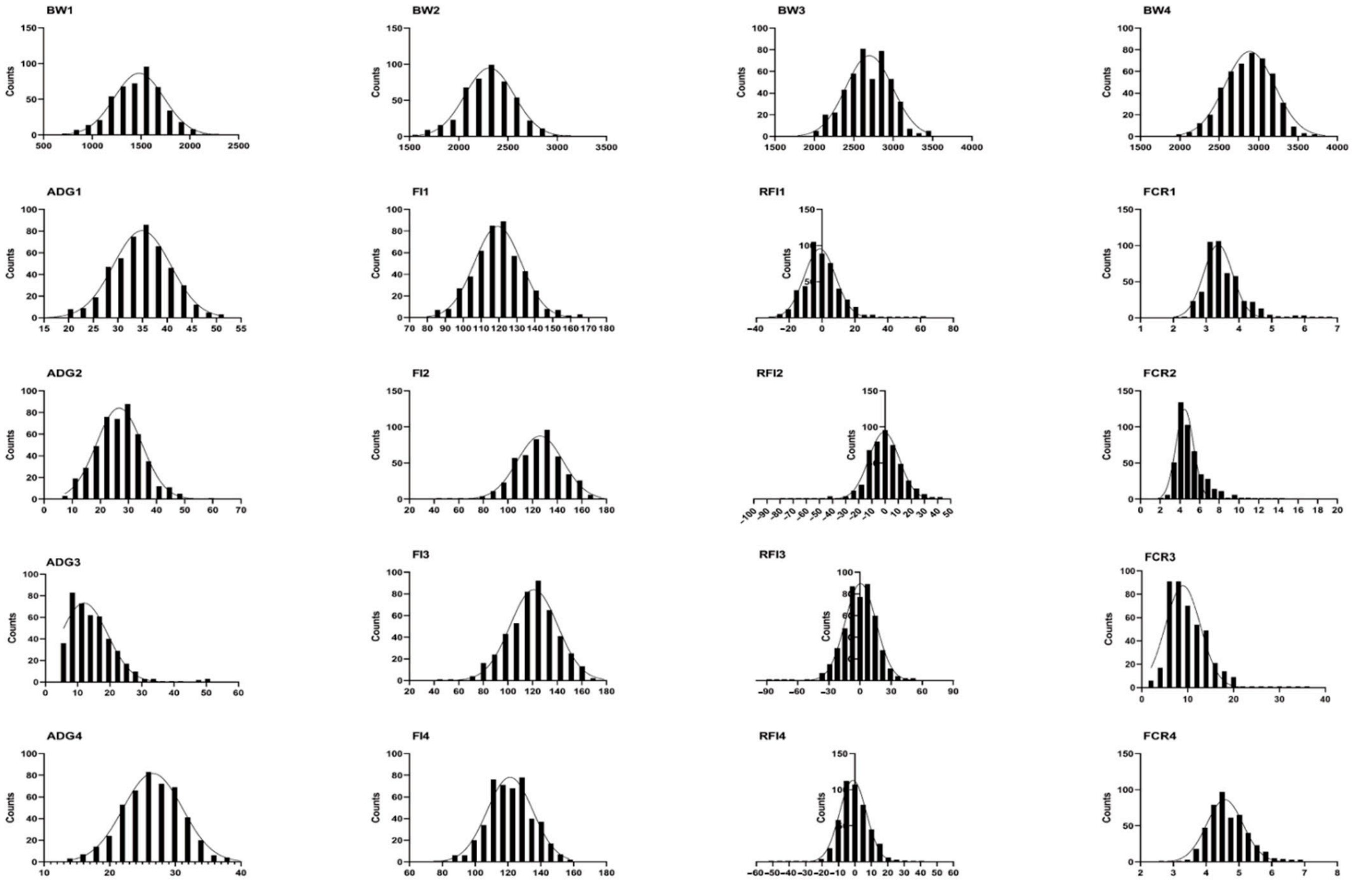

Figure S1: Histogram of normal distribution of growth traits.

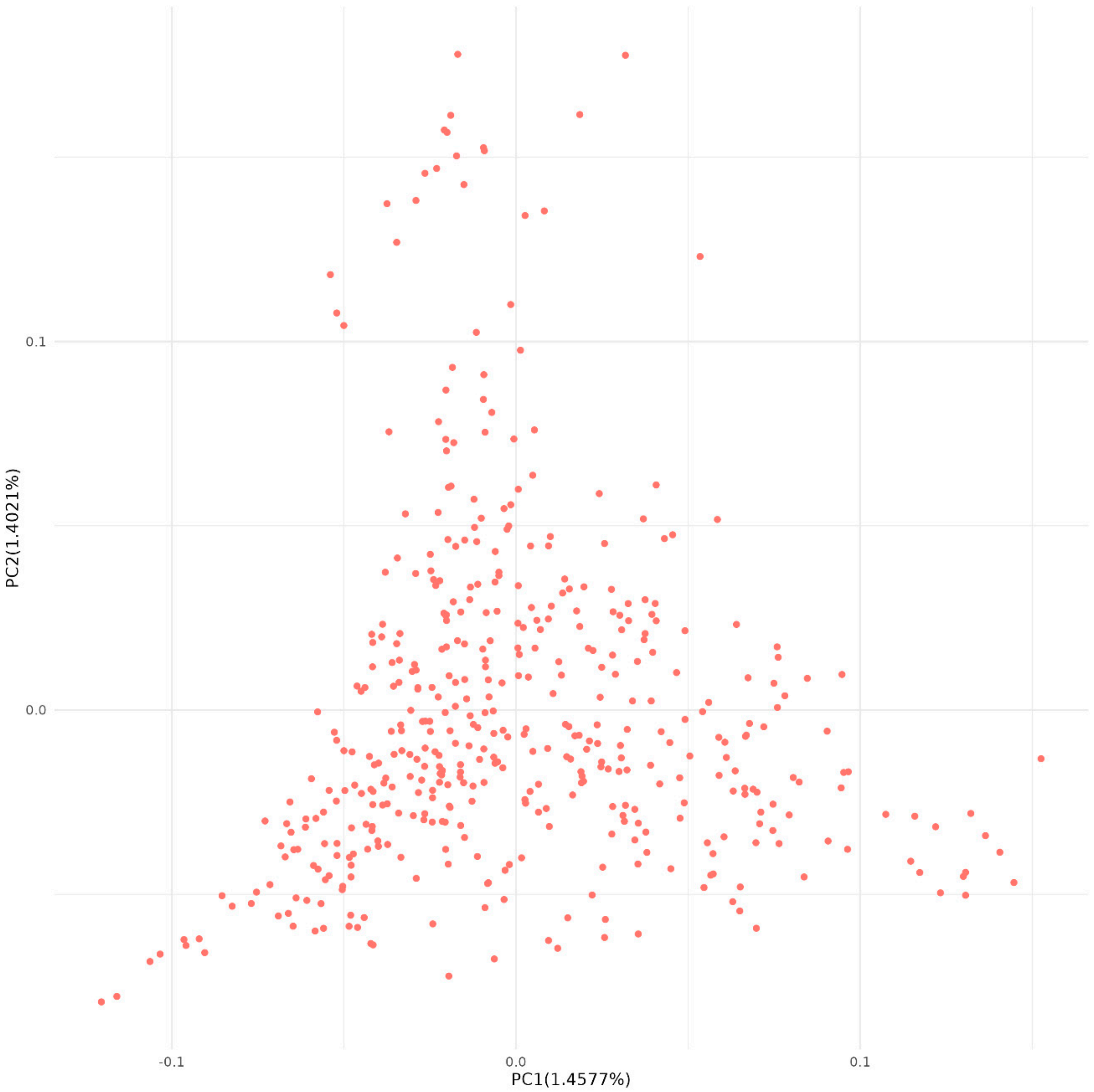

Figure S2: Principal component analysis.

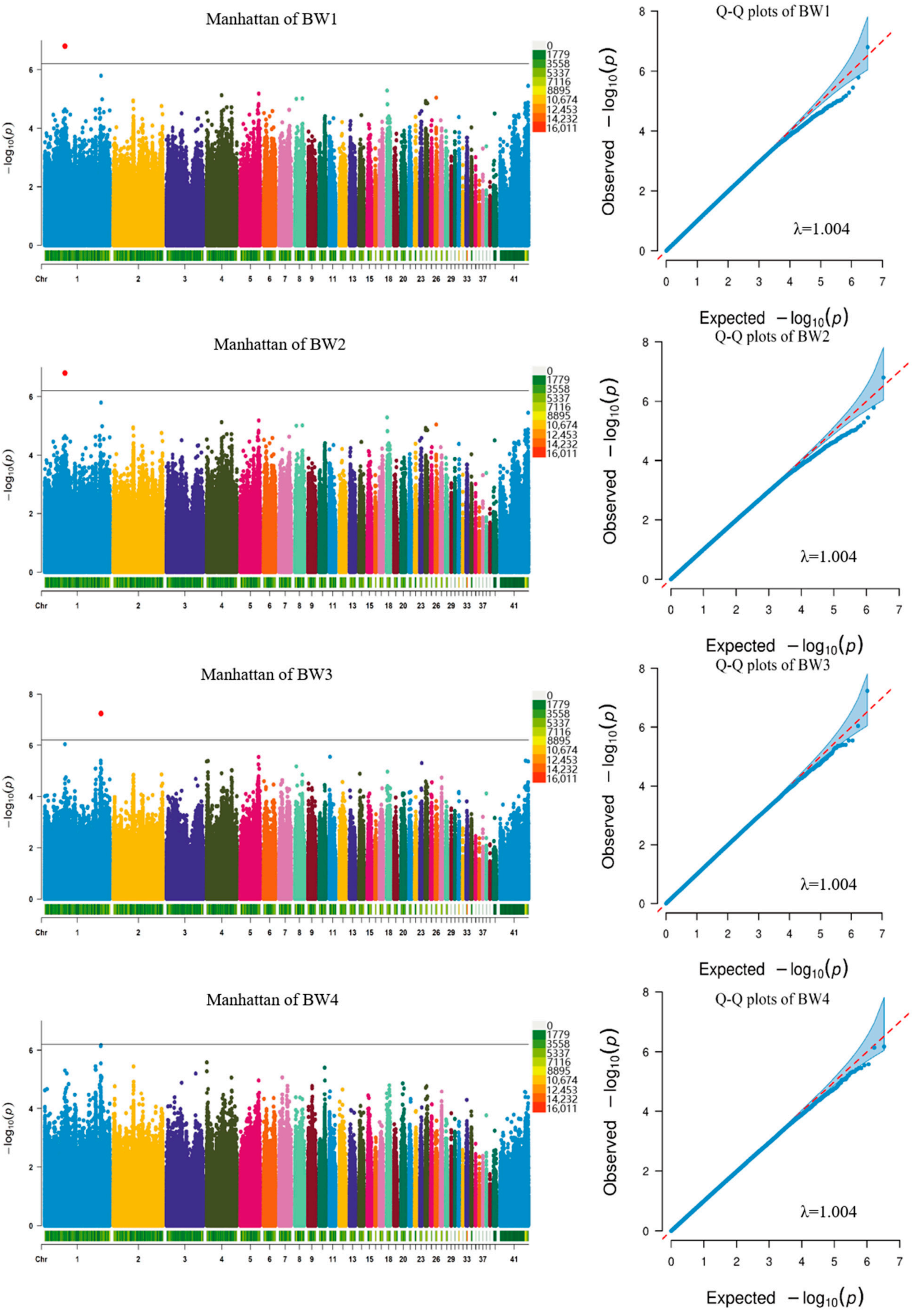

Figure S3. Manhattan chart and QQ plots of body weight GWAS in different periods (The black line in the Manhattan chart indicates the threshold, the same as below)..

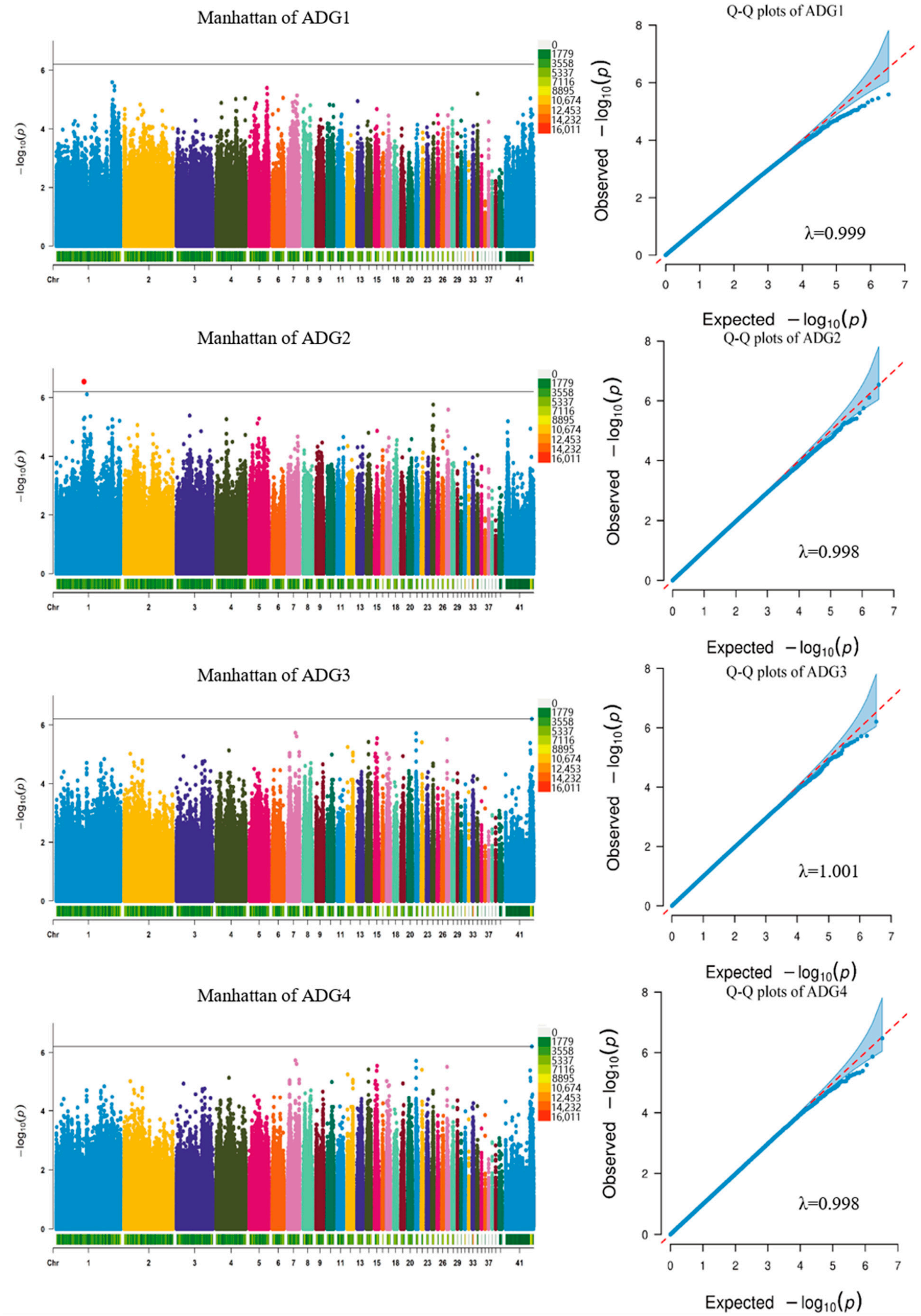

Figure S4. Manhattan chart and QQ plots of average daily gain GWAS in different periods.

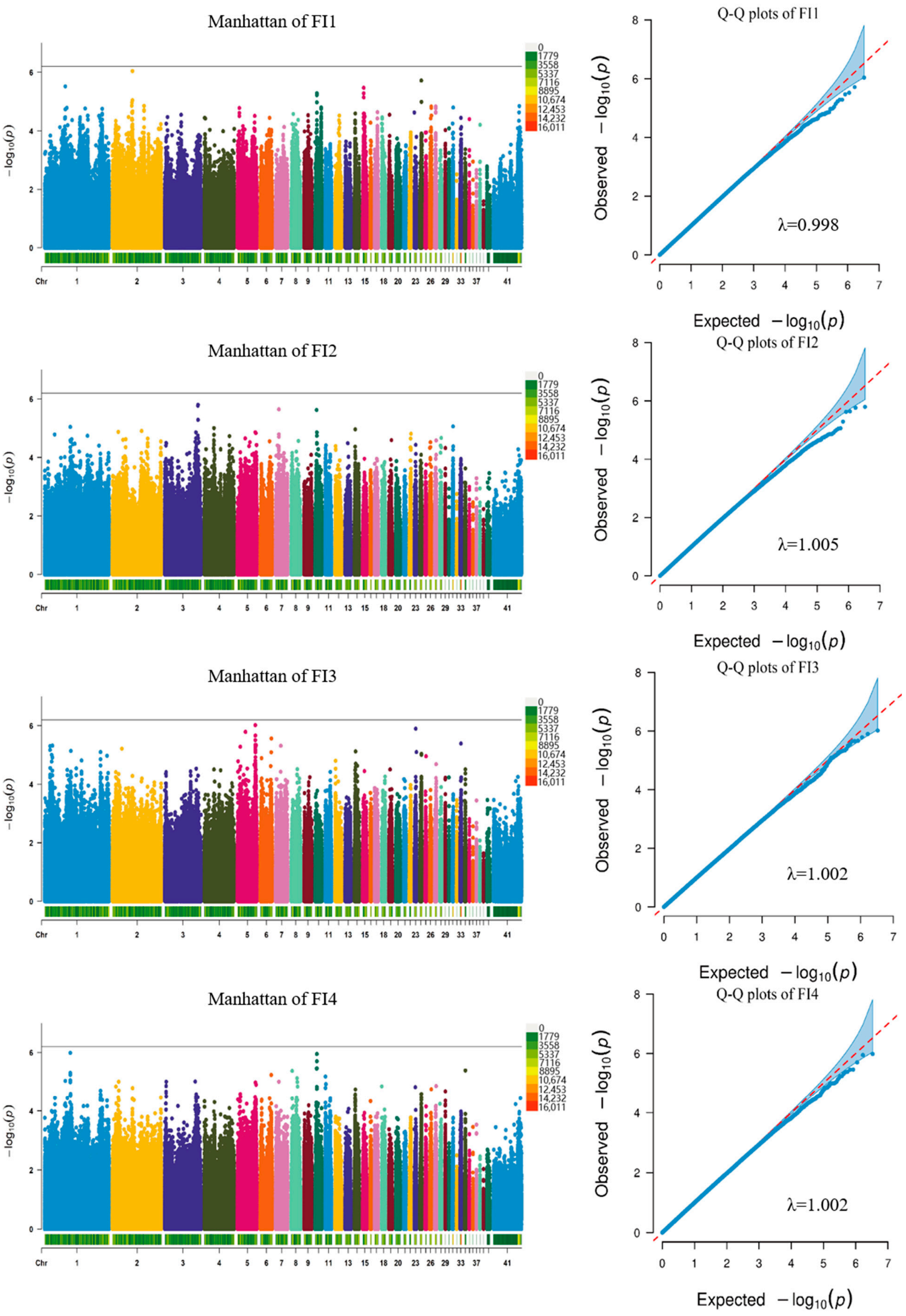

Figure S5: Manhattan chart and QQ plots of feed intake GWAS in different periods.

Table S1. Summary of quality control results of whole genome resequencing.

Chicken (n)	Raw Base (Gb)	Clean Base (Gb)	Effective Rate (%)	Q20 (%)	Q30 (%)	GC (%)
464	17.03	17.02	99.96	98.15	93.76	41.67

Raw Base (Gb): The original amount of data; Clean Base (Gb): The effective data amount after filtration; Effective Rate (%): The ratio of Clean Data obtained after filtration to Raw Data; Q20 (%), Q30 (%): The percentage of bases with Phred scores greater than 20 and 30 respectively; GC (%): The percentage of the total number of bases consisting of G and C bases to the total number of bases.

Table S3. Descriptive statistics of growth trait; n=464.

	Max	Min	Aver	Std	Var	Cv
BW1 (g)	2320.00	765.00	1465.69	255.34	65,200.06	17.42%
BW2 (g)	3160.00	1605.00	2305.51	255.31	65,182.84	11.07%
BW3 (g)	3500.00	1835.00	2682.38	286.70	82,195.05	10.69%
BW4 (g)	3830.00	1975.00	2872.91	300.96	90,579.53	10.48%
ADG1 (g/d)	51.90	16.19	34.76	5.91	34.90	16.99%
FI1 (g)	171.03	79.24	119.84	14.22	202.14	11.86%
RFI1 (g)	63.97	−28.54	−0.05	11.75	138.07	
FCR1	6.72	2.06	3.52	0.60	0.36	17.00%
ADG2 (g/d)	61.43	5.71	26.92	8.30	68.81	30.82%
FI2 (g)	176.99	45.11	125.67	18.50	342.40	14.72%
RFI2 (g)	40.79	−78.99	0.15	13.40	179.63	
FCR2	14.34	2.04	5.05	1.52	2.30	30.06%
ADG3 (g/d)	70.71	0.71	13.61	7.68	58.99	56.44%
FI3 (g)	174.01	31.50	120.10	20.02	400.72	16.67%
RFI3 (g)	54.10	−91.92	−0.18	16.09	259.02	
FCR3	147.92	1.89	12.44	11.72	137.45	94.23%
ADG4 (g/d)	41.00	13.88	26.50	4.53	20.52	17.09%
FI4 (g)	187.23	71.19	121.69	13.80	190.48	11.34%
RFI4 (g)	39.04	−52.54	−0.09	9.01	81.20	
FCR4	6.98	2.75	4.67	0.61	0.37	13.07%

Max: the maximum value; Min: minimum value; Aver: The average value; Std: Standard deviation; Var: variance; Cv: Coefficient of variation.
